# Lifelong testicular differentiation in *Pleurodeles waltl* (Amphibia, Caudata)

**DOI:** 10.1186/1477-7827-7-21

**Published:** 2009-03-05

**Authors:** Stéphane Flament, Hélène Dumond, Dominique Chardard, Amand Chesnel

**Affiliations:** 1 EA 3442 Aspects cellulaires et moléculaires de la reproduction et du développement, Nancy-Université, Faculté des Sciences, Boulevard des Aiguillettes, BP 70239, 54506 Vandoeuvre-les-Nancy, France

## Abstract

**Background:**

In numerous Caudata, the testis is known to differentiate new lobes at adulthood, leading to a multiple testis. The Iberian ribbed newt *Pleurodeles waltl* has been studied extensively as a model for sex determination and differentiation. However, the evolution of its testis after metamorphosis is poorly documented.

**Methods:**

Testes were obtained from *Pleurodeles waltl* of different ages reared in our laboratory. Testis evolution was studied by several approaches: morphology, histology, immunohistochemistry and RT-PCR. Surgery was also employed to study testis regeneration.

**Results:**

In this species, the testis is linked to the lung. This association consists of connective tissue derived from the mesorchium and the coelomic epithelium surrounding the lung and takes place at the end of larval life. This tissue contains lobules including primordial germ cells with a typical large and polylobular nucleus. The anterior part of the testis remains thin and undifferentiated while the posterior part differentiates in a large first testis lobe where spermatogenesis occurs during the first year of life. The undifferentiated status of the anterior part is attested by the lack of expression of the testis marker Dmrt1 and the meiosis entry marker Dmc1. Three-year-old *Pleurodeles waltl* possess multiple testes made up of two lobes. The second lobe appears at the caudal extremity of the first one from residual primordial germ cells located near or even inside efferent ducts in the glandular tissue that usually appears following spermatozoa extrusion. Surprisingly, in the case of surgical elimination of the anterior part of the testis, de novo spermatogenesis is stopped in the first lobe which becomes restricted to the glandular tissue. Following first testis lobe removal, the anterior part of the testis regenerates a new testis lobe, a process stimulated in the presence of DHT.

**Conclusion:**

*Pleurodeles waltl* constitute an original gonochoristic vertebrate model in which testis differentiation is observed up to adulthood.

## Background

Like in most vertebrate species, ovarian or testis differentiation in amphibians takes place from an undifferentiated bipotential organ. Two areas can be observed in the undifferentiated gonad [1]. At the periphery, the cortex corresponds to the coelomic epithelium colonized by primordial germ cells that originate from an extragonadal region. In the central part of the organ, there is the medulla derived from the mesonephros blastema. The ovary differentiates as an ovisac since germ cells stay in the cortex where they proliferate and together with somatic cells, constitute follicles whereas medulla regression generates a cavity. During testis differentiation, germ cells migrate from the cortex towards the medulla where they associate with Sertoli cells in units named cysts that are themselves included in lobules. The cortex devoid of germ cells becomes albuginea, the testis envelope. In amphibians, as well as in non-mammalian vertebrates, steroids play an important role during gonad differentiation which can be modified by experimental hormonal treatments [2,3]. Indeed, sex reversal occurs following hormonal treatment performed during the hormone-sensitive period: estradiol can induce a complete male to female sex reversal whereas dihydrotestosterone (DHT) induces a female to male sex reversal.

Among amphibians, most Caudata are atypical regarding sex differentiation because males possess two multiple testes: at least this is known in Salamandridae and Plethodontidae, two families representing 80% of Salamandroidea [4-18]. This means that in each gonad, several lobes develop successively during adult life. Among Salamandridae, the formation of these lobes has been studied in details in *Salamandra salamandra *[14]. In this species, the first testis lobe differentiates at the end of metamorphosis. The first typical spermatogenesis is observed during the third year of life: it takes place along the antero-posterior axis of the testis lobe. At the caudal part of the testis, cysts in which spermatozoa are eliminated in the efferent ducts undergo a transformation of their somatic cells: this leads to the formation of the glandular tissue which is a source of steroid hormones [19]. The glandular tissue will disappear and be replaced by small cysts containing primary spermatogonia. After a quiescent period, those cells divide to generate secondary spermatogonia leading to a second differentiated lobe that will appear at the age of 4 years at the caudal part of the testis [14]. In old males of *Salamandra*, one testis can contain up to 6 lobes.

**Figure 1:**
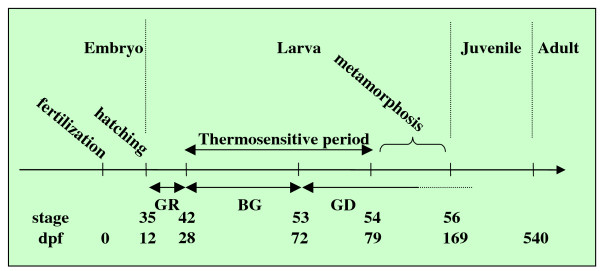
**Development of *Pleurodeles waltl***. The main developmental steps are shown and the relationship between developmental stage and age (number of days post fertilization = dpf) is indicated. Three periods of gonad formation are indicated: genital ridge (GR), bipotential gonad (BG) and gonad differentiation (GD). The thermosensitive period is the developmental window during which genetically female larvae (ZW) have to be reared at 32°C to obtain sex reversal.

*Pleurodeles waltl*, the Iberian ribbed newt found in Portugal, Spain and Northwest Africa, is a member of the Salamandridae family. Larvae possess gills and live underwater until they metamorphose into air-breathing forms possessing lungs. Sex is determined genetically (ZZ sex chromosomes in males and ZW in females) and gonad development takes place mainly during larval life (Figure 1) [3,20]. In this species, as mentioned above, sex differentiation can be modified by the environment since genetic female larvae reared at 32°C during a thermosensitive period differentiate into fertile ZW neomales (Figure 1) [21]. Here we have taken advantage of molecular tools in order to follow both the germline and somatic cell line fates in the testis in larvae, in juveniles and in adults [22]. We report that testes are associated with the lungs. This feature appears late during larval development and arises from connective tissue containing embryonic germ cells. Like other Caudata, males of *Pleurodeles waltl *possess multiple testes which differentiate during adulthood. Our experiments show that the germ cells located in the anterior part of the testis are integrated progressively into the differentiated lobe to maintain spermatogenesis. Furthermore, these cells contribute to new testis formation following castration, a phenomenon stimulated by androgen treatment.

## Methods

### Animals

*Pleurodeles waltl *larvae and adults were reared in tap water at 20 ± 2°C. Animals were fed three times a week. For larvae, developmental stages were determined by macroscopic observation according to the Gallien and Durocher development timetable [23].

Sexual genotyping was performed by electrophoretic analysis of peptidase-1 from tail biopsies as described previously [24]. For juvenile and adults, the age of each animal used in the present study was indicated in months or years (post-fertilization).

### Surgery and DHT treatments

A stock solution of benzocaine (Sigma-Aldrich, Saint-Quentin Fallavier, France) was prepared at 3% in absolute ethanol (w/v) and stored at 4°C. Animals were anaesthetized by immersion in water containing 1% (v/v) of this stock solution.

For operative procedures (either section between the posterior testis lobe and the undifferentiated anterior part or ablation of the first testis lobe), a 1.5 cm incision was made in the ventrolateral side of the abdomen and the testis was manipulated using sterile fine forceps and scissors. Animals were allowed to recover on moist paper for 24 hours. Then, they were placed in tanks containing 2 l of water supplemented or not with dihydrotestosterone (DHT). A stock solution of DHT was prepared in absolute ethanol (5 mg/ml) and stored at room temperature. For treatment, DHT was used at 400 μg/l whereas control males were exposed to ethanol. The water (with or without treatment) was renewed after each feeding.

Several months after surgery, animals were anaesthetized, sacrificed, gonads and lungs were subjected to a morphological examination before histology or molecular biology studies.

### Histology and Immunohistochemistry

Tissues were fixed in Carnoy's solution, embedded in paraffin and sectioned at 7 μm. For histological studies, sections were stained with hematoxylin-eosin-light green. Proliferating Cell Nuclear Antigen (PCNA) detection was performed using the PC10 monoclonal antibody and the LSAB2 kit (both from Dako, Trappes, France) according to manufacturer's instructions.

For BromodeoxyUridine (BrdU) incorporation, BrdU (Sigma-Aldrich) was diluted at 1 mg/ml in Steinberg's solution and 1 ml was used for intraperitoneal injection per juvenile male. After 24 hours, the animals were anaesthetized with benzocaine and testes were harvested and fixed in Carnoy's solution. Once embedded in paraffin and sectioned at 7 μm, BrdU detection was performed using a monoclonal antibody (clone BU 33, Sigma-aldrich, Saint-Quentin Fallavier, France) according to manufacturer's instructions and the LSAB2 kit.

Preparations were analysed under white illumination on a Eclipse 80i microscope (Nikon, Champigny sur Marne, France). Images were collected using LuciaG software 4.81 (Laboratory imaging) which was also used to measure morphometric parameters on germ cells as well as testis cross sections. Nuclear parameters were the perimeter and the circularity index (4πarea/perimeter^2^). When circularity index = 1, nuclei are perfectly round. Cross section parameters were area and circularity index.

### RT-PCR

The testis was separated into three parts: anterior (part linked to the lung), medium (between this anterior part and the first lobe) and posterior (the first differentiated lobe). Total RNA was extracted from each part using 200 μl of TRIzol reagent (Invitrogen Corp., Carlsbad, CA) and quantified. The detailed protocol for reverse transcription has been described previously [25]. Briefly, total RNA (1 μg) was reverse transcribed using hexamer random primers and 100 U Moloney murine leukemia virus reverse transcriptase (Invitrogen) in a total volume of 25 μL. A 2 μL aliquot of resultant cDNAs was used for PCR.

The amplification was performed with 0.5 U *Taq *DNA Polymerase (Invitrogen) in PCR buffer containing 25 mM of each deoxynucleotide triphosphate, 1.5 mM MgCl_2 _and 10 pmol of each primer in a total volume of 25 μL. Specific primers and conditions of amplification for GAPDH (Glyceraldehyde 3-phosphate dehydrogenase), VASA, DMRT1 (doublesex- and mab-3-related transcription factor 1) and DMC1 (disrupted meiotic cDNA 1) cDNAs have been described previously [22,25]. PCR products were run in a 1% agarose gel containing 0.5 mg/L ethidium bromide.

## Results

### Gonads are associated with the lungs

**Figure 2:**
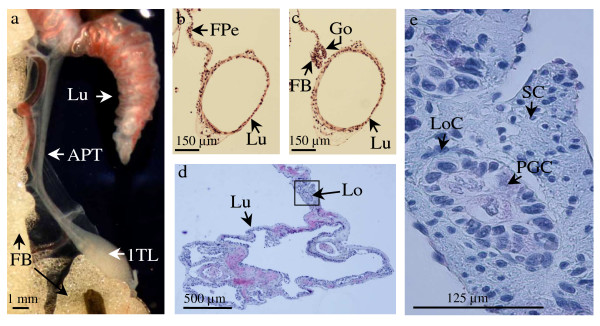
**Close association between lungs and gonads in the amphibian *Pleurodeles waltl***. (a), Anterior part of the testis (APT) associated with the lung (Lu) in a 9.5-month-old male: the enlarged posterior part constitutes the first testis lobe (1TL) and the fat body (FB) associated with the gonad is well developed. (b), Histological section of a 5-month-old male larva showing a fold of the peritoneum (FPe) linking the lung (Lu) to the mesonephros. (c), More posterior section of the animal shown in b showing the lung (Lu) separated from the fold of the peritoneum which here encloses the gonad (Go) and the fat body (FB). (d), Histological section showing the connective tissue common to the lung (Lu) and the testis in a 9.5-month-old male; lobules (Lo) containing primordial germ cells are present. (e), Enlargement of the area shown in (e); the connective link contains lobule cells (LoC) surrounding primordial germ cells (PGC) with their typical large polylobular nucleus. Outside the lobules, somatic cells (SC) possess a very small nucleus.

We observed that in males, each testis was linked to the lung of the same body side (Figure. 2a). This link was a fold of the dorsal peritoneum. In the anterior part of the abdomen, this structure surrounds the lungs whereas in the posterior part of the abdomen it surrounds the gonads and constitutes the mesorchium (Figure. 2b, Figure. 2c). At the end of larval life (at the time of metamorphosis), in the medium part of the abdomen, this fold of the peritoneum encloses both the lungs and the gonads. After metamorphosis, this region becomes a dense connective tissue. Surprisingly, in old adult animals, this structure still contains germ cells (Figure. 2d, Figure. 2e). Those cells look like the primordial germ cells that appear during the early steps of embryonic development: their nucleus is very large (perimeter = 85 ± 17 μm, n = 7) and polylobular (circularity index = 0.7 ± 0.1, n = 7).

### Testis differentiation is initially restricted to the posterior part of the gonad

At the end of larval life (stage 53, 72 dpf), germ cells leave the cortex and invade the medulla all along the cephalo-caudal axis of the gonad which is a long and thin band of tissue. At this early step of gonadal differentiation, testis and ovary can be distinguished only by histological techniques: germ cells are maintained in the cortex of the ovary but they localize in the testis medulla (not shown, see 21).

At the end of metamorphosis (stage 56, 169 dpf), a small enlargement appears at the posterior region of the organ: it constitutes the first testis lobe. The longest part of the testis is made up of a long and thin band of tissue that links the first testis lobe to the lung.

**Figure 3:**
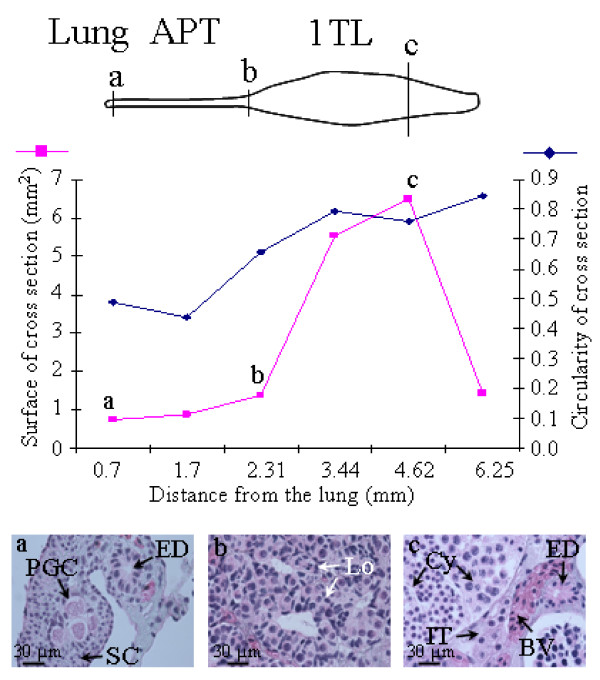
**Cephalo-caudal differences in testis differentiation**. Data is from a 1-year-old male. A chart of the testis is shown with the anterior part (APT) and the first testis lobe (1TL). The diagram shows two testis parameters measured at different points between its connection to the lung and its posterior extremity: the surface of the testis when observed in cross sections and the circularity of those sections. (a), (b) and (c) show sections stained with hematoxylin-eosin-light green, performed in three different regions indicated on the diagram and on the chart of the testis. BV = blood vessel, Cy = Cyst, ED = efferent duct, IT = interstitial tissue, Lo = lobule, PGC = primordial germ cells, SC = somatic cell.

**Figure 4:**
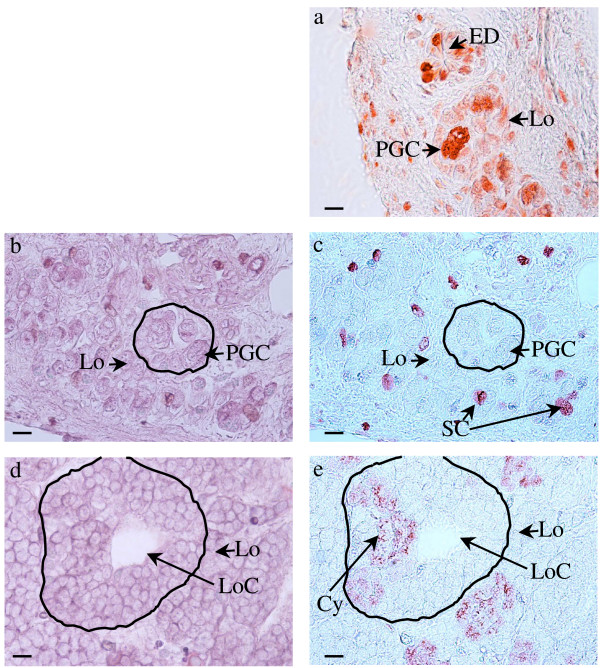
**Low proliferation rate of the primordial germ cells in the anterior part of the testis**. Data are from a 1-year-old male. (a), (c) and (e) show immunostaining; (b) and (d) show hematoxylin-eosin-light green staining of the sections shown in c and e respectively. (a), primordial germ cells in the anterior part of the testis are stained with the anti-PCNA antibody. (c), these cells do not incorporate BrdU. (e), (a) lobule at the anterior part of the first lobe, showing the synchronous proliferation of spermatogonia in a cyst attested by BrdU incorporation. Cy = Cyst, ED = efferent duct, Lo = lobule, LoC = lobule centre, PGC = primordial germ cells, SC = somatic cell.

**Figure 6:**
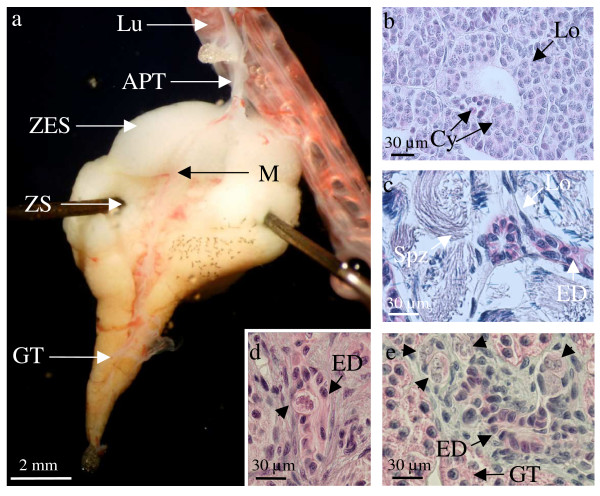
**Testis morphology in the sexually mature male**. (a), In a 2-year-old adult and sexually mature male, the anterior part of the testis (APT) near the lung (Lu) is reduced but still present. The first testis lobe possesses three parts easily identified at the morphological examination: a transluscent region at the apex corresponds to a recently appeared zone with early spermatogenic stages (ZES), a central white zone contains spermatozoa (ZS) and the posterior orange region corresponds to the glandular tissue (GT). The basis of the mesorchium (M) is observed in a depression at the surface of the testis. (b), section of the ZES showing lobules (Lo) with a central cavity whose walls contain cysts (Cy) including proliferating spermatogonia. (c), section of the ZS in which lobules contain cysts with spermatozoa (Spz). An efferent duct (ED) is also observed. d and e, sections of the glandular tissue. The glandular tissue (GT) results from the transformation of lobule somatic cells after spermatozoa extrusion. Germ cells (arrowheads) with a typical polylobular nucleus are present either in the lumen (d) or in the vicinity (e) of the efferent ducts (ED).

Between metamorphosis (6 months) and sexual maturity (1.5 – 2 years), the first testis lobe grows while the length of the anterior part of the testis linked to the lung reduces (compare Figure. 2a for a 9.5-month-old animal and Figure. 6a for a 2-year-old animal). We show the details for a one-year-old male (Figure. 3). The anterior part of the testis linked to the lung represents 30% of testis length. Cross sections performed for histology show clearly that this region is not circular (circularity index: 0.49 ± 0.02). This is due to the presence of the mesorchium and efferent ducts in the lateral part of the testis near the mesonephros (Figure. 3a). Histological analysis revealed that in this anterior region, the testis was limited by flattened epithelial cells, and medulla and cortex were clearly distinguished (Figure. 3a). The medulla contained germ cells, easily recognised by their typical large polylobular nucleus, surrounded by somatic cells. The cortex was thick and characterised by cells with a very small nucleus (Figure. 3a). In a few more anterior sections, those small cortical somatic cells were still present despite the absence of germ cells. They are the most anterior component of the testis located in the connective tissue linked to the lung. Then, we observed that the more caudal the section, the higher the number of lobules with germ cells having a polylobular nucleus. These germ cells in the anterior part of the testis were not in the G0 phase of the cell cycle since they expressed PCNA (Figure. 4a). However, they did not proliferate actively since they did not incorporate BrdU in contrast to their surrounding somatic cells (Figure. 4b, 4c).

At the beginning of the first testis lobe, sections showed numerous lobules containing cysts with proliferating germ cells (spermatogonia) with a round nucleus containing condensed chromatin. The cortex was greatly reduced in this region and the somatic cells with small nuclei were absent. The perimeter of the largest region of the first lobe was 10.37 ± 0.17 mm as determined by the analysis of cross sections stained for histology. The first testis lobe was not circular (circularity index = 0.76 ± 0.01), due to the presence of the mesorchium and efferent ducts located in a dorsal hilum. It contained lobules showing an increasing maturity throughout the cephalo-caudal axis: the cephalic part contained proliferating spermatogonia (Figure. 3b) while more caudally early spermatogenic stages were present (Figure. 3c). There was always a high synchronism of germ cells development in a cyst as illustrated by BrdU incorporation in spermatogonia (Figure. 4d, 4e).

### Differential expression of testis markers along the cephalo-caudal axis of the gonad

**Figure 5:**
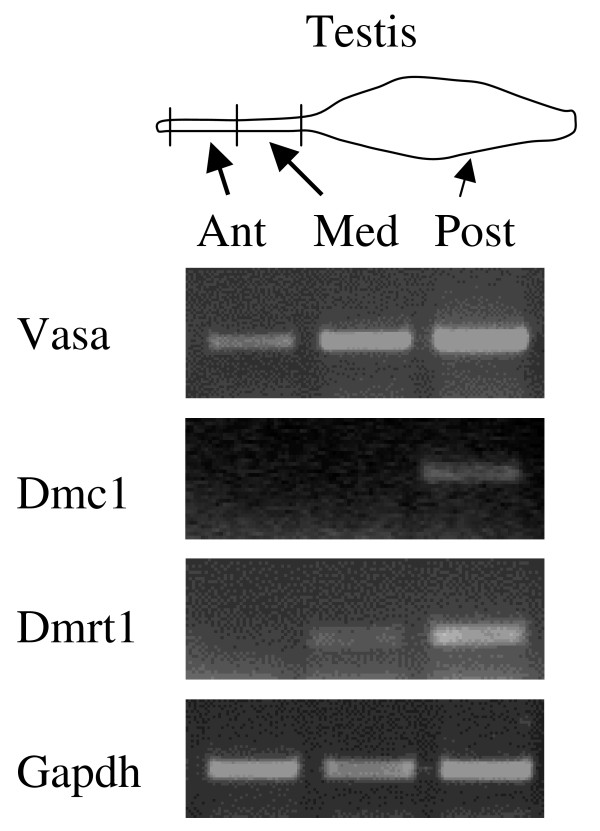
**Undifferentiated status of the anterior part of the testis linked to the lung**. RT-PCR analysis of different markers was carried out for three regions of the testis of a 9.5-month-old male: the anterior part linked to the lung (Ant), the medium part (Med) and the posterior part differentiated in a first testis lobe (Post). Marker genes: Vasa for germ cells, Dmc1 for meiosis entry, Dmrt1 for testis differentiation; Gapdh is the control gene.

Several molecular markers were studied by RT-PCR in a 9.5-month-old male (Figure. 5). First we found that the more posterior the testis region, the higher the expression of the germ cell marker Vasa. This was in agreement with our histological study showing that the number of germ cells increased along the anteroposterior axis of the gonad (see above). We also studied the expression of the meiotic recombinase Dmc1 (disrupted meiotic cDNA 1). This enzyme forms nucleoprotein complexes on single-stranded DNA that promote a search for homology and perform DNA strand exchange, the two essential steps of genetic recombination that occurs during meiosis. This meiosis entry marker was not detected in the anterior part of the testis linked to the lung (Figure. 5). This was consistent with the polylobular morphology of germ cell nucleus which is typical of primordial germ cells rather than meiotic germ cells. Dmc1 mRNA was not detected in the medium part of the testis (just in front of the first lobe) where proliferating germ cells were observed on histological sections. In contrast to the previous regions, the posterior part of the testis (first lobe) expressed Dmc1 (Figure. 5), which is in agreement with the presence of meiotic germ cells and spermatogenesis in this compartment (cf Figure. 3c). We also studied the testis specific marker Dmrt1 (doublesex- and mab-3-related transcription factor 1). This marker was not expressed in the most anterior part of the testis near the lung while it was expressed in the medium and posterior regions (Figure. 5). This suggests that the part of the male gonad located near the lung is not completely differentiated and that is consistent with the presence of a clearly identified cortex in this region (cf Figure. 3a).

### Continuous gonad differentiation leads to multiple testes

In two-year-old males, the anterior part of the testis that links the first lobe to the lung was more and more reduced (Figure. 6a). However, germ cells with a polylobular nucleus were still present in this region. The dorsal part of the testis still showed a hilum. In the first testis lobe, three areas were observed at the morphological examination: the most anterior one was translucent, the second one was white and the most posterior area was orange (Figure. 6a). The histological study shows spermatogonia, spermatocytes and spermatids in the anterior part (Figure. 6b). The second area contained only spermatozoa (Figure 6c). In other amphibians, these regions are sometimes named the immature and mature parts respectively. The orange area is the glandular tissue containing modified lobule cells appearing after spermatozoa extrusion. Surprisingly, in the glandular tissue, isolated germ cells with a polylobular nucleus were present in the vicinity of efferent ducts and sometimes in their lumen (Figure. 6d, 6e).

**Figure 7:**
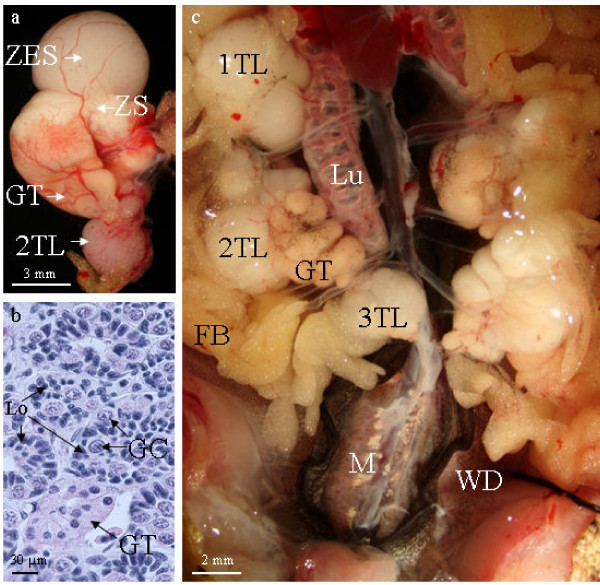
***Pleurodeles waltl *possesses multiple testis**. (a), In a three-year-old animal, a transluscent zone with early spermatogenic stages (ZES) is observed below the glandular tissue (GT). It is a second lobe (2TL) that is forming at the caudal part of the testis. (b), this new lobe contains lobules (Lo) with a central cavity and their walls are made up of cysts with proliferating germ cells (GC). (c), in a 10-year-old male 3 differentiated testis lobes (TL) are observed. The photo also shows fat body (FB), the lung (Lu), the urinary part of the mesonephros (M) and Wolfian ducts (WD).

These germ cells seemed to be quiescent up to the third year. At this moment, they become spermatogonia which together with somatic cells will generate a second testis lobe at the caudal extremity of the testis and it appears translucent (Figure. 7a). The lobules of this early new lobe showed a cavity and their wall contained very young cysts including spermatogonia (Figure. 7b). Those two lobes will be able to develop spermatogenesis with the same process as observed earlier when there was only the first lobe. Males ≥ 10 years may even possess a third testis lobe (Figure. 7c). Each lobe connected by a slender cord.

### The anterior part of the testis differentiates a new testis lobe following partial castration

**Figure 8:**
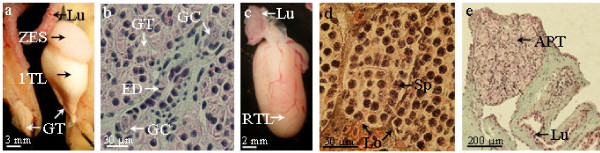
**The anterior part of the testis acts in spermatogenesis maintenance and displays high regenerating potency**. (a), 11 months after its separation from the anterior part linked to the lung (Lu), the first testis lobe is reduced to glandular tissue (GT) and there is no more spermatogenesis whereas the control testis of the same male has incorporated new germ cells in a zone with early spermatogenic stages (ZES). (b), histological section of the glandular tissue (GT) of the operated testis shown in a: germ cells (GC) with a polylobular nucleus are present in the vicinity of the efferent ducts (ED). (c), A regenerated testis lobe (RTL) near the lung (Lu) observed 8 months after the ablation of the differentiated testis lobe of a 16-month-old male. (d), Histological section of the regenerated testis lobe showing lobules (Lo) containing numerous germ cells at the beginning of spermatogenesis (Sp). (e), Histological section of the most anterior part of a regenerated testis lobe (APT) showing many primordial germ cells in the vicinity of the lung (Lu).

In order to analyse the role of the anterior region of the testis linked to the lung, it was surgically removed from 16-month-old males (n = 3) whereas the first differentiated lobe of the testis was maintained. The experiment was performed on one testis while the other one was preserved. The analyses performed on two males 11 and 13 months respectively after surgery, showed that the operated testis was dramatically reduced in size (length = 5.5 ± 0.4 mm n = 2 males *versus *17 ± 0.8 mm for the contralateral testis n = 2 males): it contained mainly the glandular tissue corresponding to lobules after spermatozoa extrusion and there was no further spermatogenesis (Figure. 8a). This glandular tissue contained isolated germ cells with a polylobular nucleus (Figure. 8b) as observed in a non-operated testis (Figure. 6e). In contrast, the control testis lobe on the contralateral side possessed glandular tissue at its posterior end but it still displayed a part containing lobules where spermatogenesis was ongoing and had acquired a new anterior part (Figure. 8a). The third male, submitted to a laparotomy 15 months after surgery, also possessed an operated testis reduced to the orange-coloured glandular tissue while a translucent small part was present at its caudal extremity, suggesting the beginning of the formation of a new lobe. The opened skin was sewn up and this animal was studied 9 months later (i.e. 2 years after surgery): the glandular tissue had been replaced by a new testis lobe as expected. This clearly showed that the anterior part of the testis near the lung was a source of germ cells for the differentiated lobe and that germ cells located in the glandular tissue contributed to the formation of a new lobe.

In another approach, the first differentiated lobe of the testis was removed while on the contralateral side of the animal, both the differentiated lobe and the anterior part of the testis linked to the lung were removed. When this experiment was performed on 16-month-old males (n = 5), eight months (n = 3) or 15 months (n = 2) after the surgery, a new testis lobe was found near the lung on the side where the anterior part of the testis had been maintained (Figure. 8c) whereas no regeneration was observed on the contralateral side subjected to a complete castration. After 8 months these new organs contained many lobules including numerous germ cells at an early stage of spermatogenesis (Figure. 8d) and in the connective tissue near the lung germ cells had proliferated (Figure. 8e). After 15 months, the regenerated testis contained a part with early spermatogenic stages, a part with spermatozoa and even glandular tissue; moreover spermatozoa were present in Wolffian ducts (not shown). So, the undifferentiated part of the testis linked to the lung has a high regenerating potency which can lead to a testis with a normal pattern of spermatogenesis.

**Figure 9:**
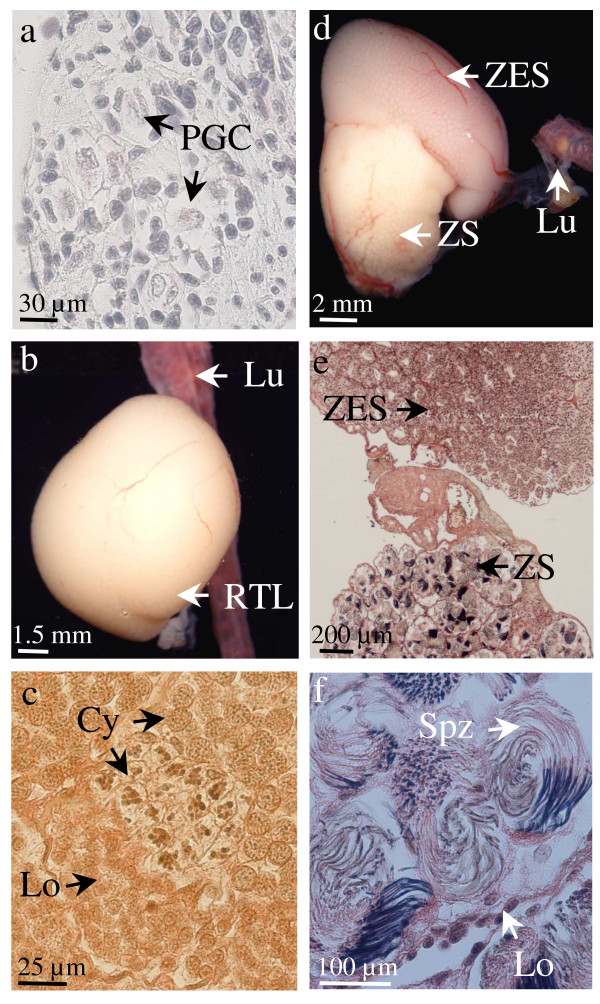
**DHT potentiates new lobe differentiation from the anterior part of the testis**. 3.3- (d-f) or 3.5-year-old males (a-c) were subjected to a bilateral elimination of the first differentiated testis lobe and then treated with DHT (b-f) or with ethanol (a = control). (a), 6 months after surgery, the anterior part of the testis linked to the lung has not yet evolved in a completely differentiated new lobe but histology reveals the presence of primordial germ cells (PGC). (b), 6 months after surgery, in case of exposure to DHT, a newly differentiated testis lobe (RTL) is observed near the lung (Lu). (c), histological section of the lobe shown in (b). A lobule containing cysts with germ cells at different stages is observed. (d), 12 months after surgery, another male exposed to DHT possesses a newly differentiated testis lobe near the lung. As illustrated by the histological section in (e), it contains an anterior zone with early spermatogenic stages (ZES) and a posterior zone with spermatozoa (ZS). F, higher magnification of the ZS showing bundles of spermatozoa (Spz) in a lobule.

Then we tested if androgens could be involved in the regulation of testis regeneration. Adult males aged between 2 and 3.5-year-old were subjected to bilateral elimination of all the testes except the anterior region linked to the lung. At the time of surgery, 2-year-old males possessed a first lobe with an anterior part containing early spermatogenic stages, a medial part with spermatozoa and a glandular part whereas 3.5-year-old males possessed the first lobe and the beginning of a second one developing caudally (see Figure 7a). One group (n = 5) was reared in water containing dihydrotestosterone at 400 μg/l (a dose known to induce functional female to male sex reversal of larvae) whereas the control group (n = 4) was reared in ethanol containing water. Surprisingly, analyses performed between 5 and 12 months post surgery failed to detect testis regeneration in control males (table 1). Nevertheless, on the histological sections we observed a higher number of germ cells with a polylobular nucleus than usually observed in non-operated animals near the lung (Figure. 9a). In contrast, DHT-treated males possessed a regenerated testis that was easily observed near the lung after dissection (table 1). Six months after surgery, the newly differentiated lobe contained only one zone with early spermatogenic stages (Figure. 9b, 9c). One year post-surgery, the regenerated testis had reached 1.5 cm in length and possessed two parts: the anterior part was a zone with early spermatogenic stages whereas the posterior part contained spermatozoa (Figure. 9d, 9e, 9f). This experiment strongly suggests that androgens could regulate the regeneration process and probably new lobes differentiation in a more physiological context.

**Table 1:** DHT promotes testis regeneration

**Treatment and number of animals**	**Age at surgery (years)**	**Duration of treatment (months)**	**Testis regeneration**
**First testis lobe removal**	2	5	No
N = 4	3.5	6	
	3.5	6	
	3.5	12	
			
**First testis lobe removal + DHT**	2	5	Yes
N = 5	3.5	6	
	3.5	6	
	3.3	12	
	3.3	12	

## Discussion

### Gonads linked to the lungs

In *Pleurodeles waltl*, gonads (testes and ovaries) are associated with the lungs. Lungs are paired ventral derivatives of the foregut and their development depends on interactive signalling between the endodermal epithelium and mesenchyme derived from splanchnic mesoderm [26-28]. Gonads arise caudally as a thickening along the ventromedial side of the mesonephros. Thus, lungs and gonads show different development process, localization and function and they are never associated. This is obvious in mammals where the diaphragm separates the rib cage containing lungs and the abdomen containing gonads but this is also observed in birds and reptiles which do not possess a diaphragm, as well as in anuran amphibians like frogs or toads that do not have a rib cage. The presence of a link between gonads and lungs cannot exist in Plethodontids that are lungless salamanders. Such a link has not been described in studies about testis development or spermatogenesis in *Pleurodeles waltl *although the presence of primordial germ cells at the anterior part of the testis is well known [29,30]. This link has not been described in any other Caudata species [18]. Studies on lung development in Caudata also do not describe any association with the gonads. We have had the opportunity to study two other male urodele amphibians from histological sections available in the laboratory: *Ambystoma mexicanum *(Ambystomatidae) and *Ichthyosaura *(= *Mesotriton *= *Triturus*) *alpestris *(Salamandridae). In neotenic larvae of *Ambystoma mexicanum*, the lungs display more compartments than those of *Pleurodeles waltl *whereas *Ichthyosaura alpestris *possesses smooth-surfaced lungs [31,32]. Our observations indicate that a connective link between lung and testis is also observed in both species but it does not contain germ cells as described in *Pleurodeles waltl*. We found only one sentence in a short report about *Triturus cristatus*: "In case of bilateral castration a few islands of germ cells intimately linked to the lung or to the kidney cannot be extirpated" [33]. This suggests that the presence of germ cells in the link between gonad and lung might be restricted to a few species, but a detailed study of other species is required to get more information.

### Regionalized differentiation of the testis

The second interesting finding reported in our study is the fact that testis differentiation in *Pleurodeles waltl *is regionalized. Although the migration of the germ cells from the cortex towards the medulla occurs all along the cephalo-caudal axis of the testis, in juvenile animals, the differentiation will appear first in the posterior region that evolves in a first testis lobe where spermatogenesis will take place. Our results show clearly that in the anterior part of the testis, germ cells have the morphological features of primordial germ cells (large polylobular nucleus) rather than those of spermatogonia (round nucleus). This is consistent with previous reports on *Pleurodeles waltl *indicating the presence of primordial germ cells at the anterior part of the testis [30]. After a 24 hour exposure to BrdU, these cells have not incorporated the thymidine analogue whereas it was incorporated in somatic cells in the same area and in numerous spermatogonia in the first testis lobe. These germ cells located in the anterior part of the testis linked to the lung are not out of the cell cycle since they express PCNA. Thus, they just do not proliferate actively. This regionalization is not a unique feature of this animal since histological studies have shown in numerous Caudata that the cephalic part of a testis lobe contains primordial germ cells whereas the caudal part contains spermatozoa. In other amphibians, these regions are sometimes named the immature and mature parts respectively [14,34,35]. Such a regionalization is very interesting for the study of spermatogenesis and its regulation [35,36]. Nevertheless, the molecular markers analyzed in our study to better characterize testis differentiation status have been used only in a few species. Germ cells in the anterior part of the testis have not entered meiosis as confirmed by the absence of expression of the recombinase Dmc1 [37]. In the newt *Cynops pyrrhogaster*, Dmc1 expression was found to be absent from spermatogonia whereas the maximal expression was observed in primary spermatocytes at the leptotene-zygotene stage [38]. Despite the presence of surrounding somatic cells and differentiated efferent ducts, this part of the testis does not express the testis specific marker Dmrt1 [20]. The number of germ cells and the differentiated status of the testis increase along the cephalo-caudal axis from the lung to the first differentiated lobe. It has been demonstrated recently that retinoic acid was involved in meiosis entry in mammals [39]. Although the evidence of a similar process in amphibians has not yet been reported, it might be interesting to determine why these cells in the anterior part of the testis do not respond to signals triggering meiosis entry (perhaps retinoic acid) whereas most posterior germ cells do.

### Undifferentiated part of the testis and spermatogenesis

We have shown that the first testis lobe is reduced to glandular tissue when the first differentiated lobe is separated from the anterior part of the testis. This demonstrates clearly that the undifferentiated part of the testis near the lung is a source of germ cells which enter progressively into the first testis lobe together with somatic cells to constitute lobules and cysts. This is in accordance with the well known increasing maturity of the lobules throughout the cephalo-caudal axis of the testis [30]. This is also in agreement with the idea that in Caudata, each annual recrudescence of spermatogenetic activity arises from the primordial tissue in the testicular cords at the anterior end of each lobe [18]. Thus, lobules in the testis of Caudata are transient structures that are replaced with new generations of lobules after sperm liberation (spermiation).

The high potency of the anterior part of the testis linked to the lung to differentiate new testicular structures has been demonstrated in our study by the elimination of the first differentiated testis lobe, triggering the formation of a new testis lobe in which a complete spermatogenesis can occur. A similar result was obtained in *Cynops pyrrhogaster *and *Triturus cristatus *and described as testis regeneration [11,33]. In the experiments performed in *Triturus cristatus*, regenerates were studied up to 21 months after the bilateral castration and several oocytes could be found in the gonad [33]. In our study, animals were not reared more than 15 months post-castration, but we did not observe oocytes on histological sections and there was no intersexuality.

The regenerating process appeared slower in older males (2- to 3.5-year-old instead of 16-month-old). However, this slower process allowed us to show very clearly a stimulation by DHT when added to the rearing water. This suggests that *in vivo *changes in steroid synthesis could be associated not only to testis regeneration but also to lifelong testis differentiation. However, little is known about androgen synthesis in *Pleurodeles waltl*. A measure of sex steroid levels in the plasma by radio-immunoassay showed two annual peaks in october-november and in march with testosterone being the major steroid in addition to DHT and androstenedione [40]. However, this study showed individual fluctuations and the age of the males is unknown (the author just indicates the use of "sexually mature males"). The author also reports that in contrast to testosterone, the levels of DHT are essentially unaffected by castration: 60 to 100% of initial DHT levels versus 0.7 to 7% of initial testosterone levels were found 11 days post-castration [40]. Together with the fact that DHT was present only at very low concentrations in the testis [41], this result suggests that DHT could be produced from an organ other than the testis. This is consistent with our finding showing that DHT can stimulate the differentiation of new testicular structures even after removal of the first testis lobe. The fact that DHT stimulates testis regeneration is in agreement with data from the Japanese red-bellied newt in which plasma DHT levels peak at the resumption of spermatogenesis [42]. Of course we cannot exclude that in addition to DHT, other factors play a part in the differentiation of a new testis lobe. For instance follicle-stimulating hormone and epidermal growth factor have been shown to regulate spermatogonial proliferation in the newt *Cynops pyrrhogaster *[35,43].

### The multiple testes

Our study also shows that in *Pleurodeles waltl*, males possess multiple testes. Such an organ is a common feature of most Caudata belonging to the Salamandroidea order. Among the Salamandridae family, it was described for instance in various genus of the Pleurodelinae subfamily (*Triturus cristatus, Ichthyosaura alpestris, Lissotriton helveticus (= Triturus palmatus*) [5,6], *Triturus marmoratus *[44], *Notophthalmus (= Diemyctylus) viridescens, Taricha torosa *(= *Diemyctylus torosus *= *Triturus torosus*) [7,12], *Cynops pyrrhogaster *[11], *Euproctus platycephalus (= rusconi), Tylotriton verrucosus *and *Calotriton (= Euproctus) asper *[15] as well as in the Salamandrinae subfamily (*Salamandra salamandra *(= *maculata*) [5,14]. Multiple testes are also present in the Plethodontidae family, both in the desmognathinae (*Desmognathus fuscus *[7,13], *D. monticola, D. ochrophaeus and D. quadramaculatus *[17]) and the Plethodontinae subfamilies (*Eurycea bislineata *[16], *Chiropterotriton mosaueri *[45]). A similar testis structure seems also to exist in the Gymnophiona *Ichthyophis tricolor *and *Uraeotyphlus cf. narayani *[46].

In *Pleurodeles waltl*, the way of formation of the new testis lobe which appears at the posterior extremity of a previous one, seems to be identical to what has been described in *Salamandra salamandra *[14] or in *Desmognathus fusca *[7]. The germ cells of the new lobe are derived from primordial germ cells located in the glandular tissue at the posterior end of the preexisting lobe. In the glandular tissue of *Pleurodeles waltl*, we found primordial germ cells in the connective tissue surrounding efferent ducts and sometimes in the lumen of those ducts. This observation confirms previous reports [30,47]. However, in the part of *Pleurodeles waltl *testis containing cysts with spermatozoa, we observed rarely primordial germ cells. The hypothesis of a migration along the efferent ducts from the anterior part of the testis is interesting but we could not test it. However, germ cells could also have another origin. Indeed, from experiments using estradiol benzoate treatment of adult males of *Cynops pyrrhogaster*, it has been proposed that germ cells could differentiate from the columnar epithelium surrounding the slender cords which connect testis lobes [48].

We have no information about the origin of somatic cells in the new testis lobe of *Pleurodeles waltl*. It has been proposed that in the early glandular tissue, after spermiation, Sertoli cells degenerate and they could differentiate into duct cells. This is suggested by the presence of a common basal lamina [49] and the expression of the WT1 gene [50]. The fact that Sertoli cells and duct cells have similar lectin-labeling patterns is also in agreement with a common origin for Sertoli cells and duct cells [30,47].

Besides, the factors that trigger the formation of a new lobe are unknown although androgens seem to be good candidates as suggested from our castration experiments.

## Conclusion

Like other Caudata, *Pleurodeles waltl *constitutes an original gonochoristic vertebrate model in which testis differentiation is observed up to adulthood. Indeed, the differentiation of gonads at adulthood occurs mainly in hermaphroditic teleost fish which are either protandrous (e.g; gilthead seabream) or protogynous (e.g. honeycomb grouper) [51,52]. *Pleurodeles waltl *might be used for the study of factors required to maintain primordial germ cells in adults as well as factors triggering their differentiation. This could shed light on the highly debated recent finding of germline stem cells in the mammalian ovary [53].

## Competing interests

The authors declare that they have no competing interests.

## Authors' contributions

SF designed the study, performed surgical experiments, organ collection, analyzed histological sections and drafted the manuscript. HD realized molecular biology analyses. DC performed steroid treatments and contributed to histological analysis. AC obtained data about molecular tools and critically reviewed the manuscript. All authors read and approved the final manuscript.
